# Acute Fatal Gastric Dilatation and Volvulus in a Captive Adult Linnaeus’s Two-Toed Sloth (*Choloepus didactylus*) in Amazon Biome

**DOI:** 10.3390/ani14233527

**Published:** 2024-12-06

**Authors:** Hanna Gabriela da Silva Oliveira, Cinthia Távora de Albuquerque Lopes, Letícia Yasmin Silva Correa, Roberta Martins Crivelaro Thiesen, Rodrigo Otavio Silveira Silva, Francisco Alejandro Uzal, Sheyla Farhayldes Souza Domingues, Felipe Masiero Salvarani

**Affiliations:** 1 Instituto de Medicina Veterinária, Universidade Federal do Pará, Castanhal 68740-970, PA, Brazil; hanna.oliveira@castanhal.ufpa.br (H.G.d.S.O.); cinthia@ufpa.br (C.T.d.A.L.); leticia.correa@castanhal.ufpa.br (L.Y.S.C.); roberta.crivelaro@gmail.com (R.M.C.T.); shfarha@ufpa.br (S.F.S.D.); 2 Laboratório de Bacterioses e Pesquisa da Escola de Veterinária, Universidade Federal de Minas Gerais, Belo Horizonte 31270-901, MG, Brazil; rodrigo.otaviosilva@gmail.com; 3 School of Veterinary Medicine, University of California, Davis, CA 95616, USA; fauzal@ucdavis.edu

**Keywords:** sloth, stomach, gastric torsion, *Clostridium perfringens* type A

## Abstract

This study aims to report the dietary and daily management, clinical signs, complementary exams, and pathological findings related to an acute and fatal case of gastric dilatation and volvulus (GDV) in a captive Linnaeus’s two-toed sloth (*Choloepus didactylus*) in the Amazon Biome. An adult female sloth, rescued after being electrocuted, was housed at the Wildlife Section of the Veterinary Hospital (WSVH) of the Institute of Veterinary Medicine (IVM) at the Universidade Federal do Pará (UFPA). It was fed a diverse diet that included animal protein, fruits, vegetables, and greens, with vitamin and mineral supplementation. After five years, the sloth was found in its enclosure hyporesponsive, dehydrated, hypothermic, and hyperventilating, with an abdominal dilation of firm consistency. During emergency care, the animal died. Fecal samples collected two days before death were positive only for *Clostridium perfringens* type A. Necropsy findings included dilatation of the squamous gastric compartment due to gas accumulation, with necrotic and hemorrhagic mucosa, a 180–270° volvulus obstructing the gastroesophageal region, and intestinal mucosal hyperemia. Histopathology revealed marked congestion with hemorrhage and gastric mucosal necrosis, along with tertiary lymphoid follicles in the submucosa. To the authors’ knowledge, this is the first report of GDV in a sloth in Brazil. Further research is needed to develop appropriate approaches to GDV in captive sloths.

## Simple Summary

The Brazilian Amazon is home to the largest species of the Megalonychidae family, *Choloepus didactylus*, the Linnaeus’s two-toed sloth (LTT). Due to their diverse diet, they adapt well to captivity conditions. However, digestive disorders associated with stress and prolonged captivity are commonly reported. Gastric dilatation and volvulus (GDV), characterized by the rotation of the stomach around its axis, has been previously documented in LTTs in international institutions. Therefore, it is important to understand the potential contributing factors and etiological agents involved in its pathogenesis, especially as urban and agricultural expansion in the Amazon Biome may increase the incidence of this species requiring care and remaining in captivity. This report describes an acute and fatal case of GDV in an adult captive LTT, which, to our knowledge, has not been previously reported in the Brazilian literature. Feeding and daily management are described, including clinical signs, complementary examinations, pathological findings, and discussion of potential contributing factors.

## 1. Introduction

The Brazilian Amazon is home to the largest species of the family Megalonychidae, from the superorder Xenarthra, *Choloepus didactylus*, commonly known as the Linnaeus’s two-toed sloth (LTT) [1,2]. Although it is prominently present in northern Brazil, this species is not endemic to the country, as it also occurs in Colombia, Venezuela, the Guianas, Ecuador, and Peru [2]. The two-toed sloth is characterized by two clawed digits on each forelimb, with a body mass ranging from 4 to 8.4 kg and a length between 60 and 86 cm [1]. Additionally, it has solitary, nocturnal, and arboreal habits, spending most of its time in trees with climbing plants for resting. Its fur is home to symbiotic algae that assist in camouflage and protection against potential predators [1,3].

Sloths are considered herbivores, with their diet mainly consisting of leaves, twigs, shoots, and fruits. However, the LTT occasionally feeds on symbiotic algae, eggs, and insects [3,4]. Its large, chambered stomach houses bacteria that aid in digestion through foregut fermentation [1]. It is also believed that the species’ low metabolic rate, low food intake, and infrequent defecation reduce their energy requirements, allowing them to subsist on low-calorie diets, resulting in long periods of inactivity and slow movements [5].

These animals are ecologically important, contributing to soil nutrient cycles and pest control, and due to their diverse diet, they adapt well to captivity [5,6]. However, digestive disorders associated with stress and time in captivity are commonly reported in these species, including constipation and bloat [3,5,7,8]. In this context, gastric dilatation and volvulus (GDV), characterized by the rotation of the stomach around its axis with multifactorial etiology, has been reported in this species in institutions in the United States, Canada, and Germany [9,10]. GDV leads to multiple organ dysfunction syndrome (MODS) and is rapidly fatal, highlighting its importance for LTT conservation in captivity [9,10].

Although the LTT is listed as “least concern” on the IUCN Red List of Threatened Species, urban and agricultural expansion associated with intense deforestation and fires in the Amazon Biome may increase the number of cases seen in Wildlife Rehabilitation Centers (CETAS) and veterinary hospitals. Therefore, a better understanding of the potential contributing factors and etiological agents involved in the pathogenesis of gastrointestinal disorders in these captive animals is necessary [6,8,10,11].

Thus, the aim of this article is to report an acute and fatal case of GDV in an adult LTT (*C. didactylus*) that had been kept in captivity for five years. Details of its dietary and daily management, clinical presentation, complementary examinations, and pathological findings are provided, alongside a discussion of possible contributing factors. This is the first case report of this condition in *C. didactylus* in Brazil within the Amazon Biome, with the goal of contributing to emergency care and stabilization protocols for these animals in captive conditions.

## 2. Materials and Methods

### 2.1. Ethical Approvement

This study was authorized by the Animal Experimentation Ethics Committee (CEUA) of the UFPA under protocol number 8888280618 (ID 002193).

### 2.2. Case Report

A free-ranging adult female Linnaeus’s two-toed sloth (*Choloepus didactylus*), weighing 6.15 kg, was rescued after an electric shock by agents from the Municipal Environment Department (SEMMA) and subsequently referred to the WSVT of the IVM at the UFPA.

During the initial clinical evaluation, the patient presented with burns on the right lateral side, blistering, and necrotic ulcers on the thoracic and pelvic limbs, as well as an open fracture in the left radio-ulnar-carpal joint with compromised skin, muscle, and bones due to necrosis. Surgical procedures were performed for stabilization, including amputations of the left thoracic limb (LTL), right thoracic limb (RTL), and right pelvic limb (RPL), along with wound debridement. In addition, medication, nutritional, and occupational therapies were administered [12].

After five months of treatment, the animal showed clinical improvement and remained in WSVT for five years due to difficulties finding a conservation facility for relocation. In the first days of hospitalization, the animal was fed using a 20 mL syringe with a paste-like consistency, and food strips were also offered, consisting of carrots, kale, bananas, mangoes, apples, watermelon, melon, and various juices. After three months, the patient began eating spontaneously, approaching the food dish directly. The diet was gradually altered to a fixed diet, offered twice daily.

In the morning, animal protein, fruits, and vegetables were provided in strips, while in the afternoon, a paste-like mixture was prepared and offered, consisting of fruits, vegetables, greens, animal protein, and water (Table 1), yielding approximately 400 mL. Additionally, 0.2 g of an animal supplement (Aminomix^®^ Pet) was incorporated into both diets, which the animal accepted and chewed well. Occasionally, embaúba leaves and other fruits (guava, grapes, pineapple, avocado, mango, melon), vegetables (beetroot, pumpkin, and sweet potato), and greens (kale) were also provided as environmental enrichment once a week. Water was available ad libitum.

Independent analyses of diet samples, based on the Brazilian Food Composition Table (TACO), regarding moisture, energy, protein, carbohydrates, lipids, fiber, calcium, and magnesium, demonstrated that the diet adequately met the species’ nutritional requirements. Twice weekly, a multivitamin supplement (Glutamina^®^) was administered orally at a dose of 0.1 mL/kg. The patient was weighed daily, with an average and standard deviation of 4.7 kg ± 0.7 kg during her stay at WSVT.

Throughout the five years, the animal was housed in a specific area for wild mammals, in a transport box (84 cm × 73 cm × 60 cm) with exposure to natural ultraviolet light, lined with soft fabrics and pillows. The enclosure was cleaned daily and washed and dried weekly, and the fabrics and pillows were replaced regularly. The animal was kept isolated from other species, without direct contact with other animals. The ambient temperature ranged from 23 °C to 35 °C, with an average humidity of 70%.

The animal was monitored daily for behavior and body temperature. Behavior assessment included visual analysis of activity or agitation levels, as well as facial expressions and possible vocalizations indicative of discomfort. Body temperature was measured from different regions, such as the pseudo-cloaca, using a digital clinical thermometer (Termo med 10^®^), and from the snout area using a non-contact infrared thermometer (G-tech^®^, Accumed, Belém, Brazil). The average and standard deviation of the temperatures were 34.3 ± 0.7 °C and 35.1 ± 0.6 °C, respectively.

**Figure 1:**
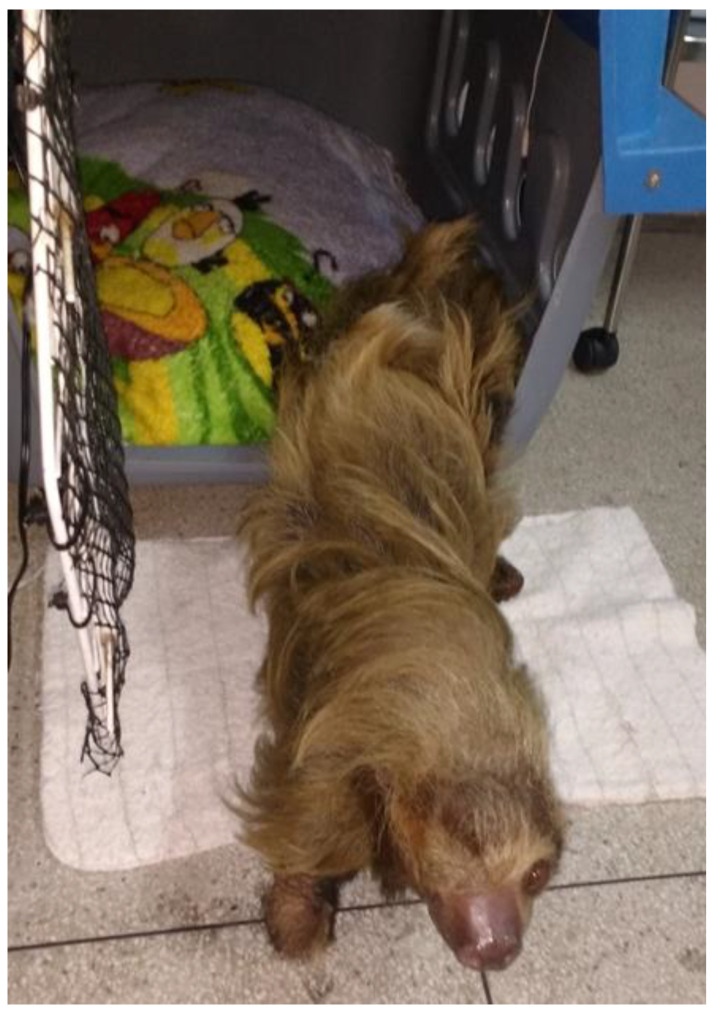
The patient, supported on the stumps of fully healed amputated limbs, is seen exiting the transport crate where it had been housed.

The patient was able to move freely using the stumps of the amputated limbs for support (Figure 1) and could rotate around her axis, sometimes lying on her back. However, she was unable to fully hang from trunks, which were occasionally added to her environment as enrichment, spending most of her time in direct contact with the ground. She was taken outdoors daily to sunbathe in a protected grassy area for 15 to 20 min, usually after feeding, to stimulate defecation, urination, and gas release. The sessions were supervised by a team of three alternating veterinarians. During sunbathing, the patient was occasionally observed engaging in geophagy.

The animal maintained an average urination frequency of every two days and defecation every five days, both of which remained consistent throughout her hospitalization, with urine and feces exhibiting normal characteristics for the species. Occasionally, during daily handling, abdominal distension was observed. Abdominal ultrasonography was performed using the Focused Assessment with Sonography for Trauma (FAST) method to evaluate for any abnormalities. When gas accumulation was detected, which could indicate bloat, simethicone (Luftal^®^, Reckitt, São Paulo, Brazil) was administered orally at a dose of 12 mg/kg.

Fecal samples were collected and analyzed every six months for parasites, with negative results using direct and centrifugal flotation methods [13,14] at WSVT. Bacteriological cultures for *Salmonella*, *Shigella*, *Plesiomonas*, *Edwardsiella*, *Aeromonas*, *Yersinia*, *Campylobacter*, *Clostridium perfringens*, and *Clostridioides difficile* were negative, as tested by the Preventive Veterinary Medicine Laboratory at UFPA’s IVM. A fecal sample collected two days before the animal’s death was sent to the Federal Agricultural Laboratory (FAL) of the Brazilian Ministry of Agriculture, Livestock, and Supply for mass spectrometry analysis using MALDI-TOF (Vitek^®^ MS, bioMérieux, Marcy l’Etoile, France) to detect *Salmonella*, *Shigella*, *Plesiomonas*, *Edwardsiella*, *Aeromonas*, *Yersinia*, *Campylobacter*, *Clostridium perfringens*, *Clostridioides difficile*, and *Sarcina* spp. [15]. Additionally, the sample was sent to the Anaerobic Laboratory at the Federal University of Minas Gerais (UFMG) for culture and molecular analysis (Thermal Cycler Px2, Thermo Fisher, Waltham, MA, USA) to identify *C. perfringens* and *C. difficile* [16,17]. Another parasitological examination was also conducted at WSVT [13,14].

On the day before death, the animal showed reduced appetite, leaving part of its food uneaten, but no other abnormalities were noted. On the day of death, the animal was found in its enclosure in a prone position, unresponsive, with increased capillary refill time and skin turgor, pale mucous membranes, a measured temperature below 32 °C using a digital clinical thermometer (Termo med 10^®^), muffled heart sounds on auscultation (Littmann Classic III^®^), and hyperventilation with a respiratory rate of 36 breaths per minute with shallow abdominal movements. Abdominal distension was noted, with a firm but flexible consistency upon palpation.

The patient was immediately given emergency care. A heater was used to attempt to stabilize body temperature, 50 mL of fluid therapy (NaCl 0.9%) was administered subcutaneously for electrolyte replenishment, and oxygen therapy began with 60% oxygen. However, during the procedure, the animal experienced cardiorespiratory arrest, and despite attempts at cardiopulmonary resuscitation, it was unsuccessful.

## 3. Results

### 3.1. Microbiological, Parasitological, and Molecular Findings

Fecal samples collected two days before the animal’s death and sent to the FAL for mass spectrometry testing were negative for *Salmonella*, *Shigella*, *Plesiomonas*, *Edwardsiella*, *Aeromonas*, *Yersinia*, *Campylobacter*, and *Clostridioides difficile* but positive for *Clostridium perfringens.* The sample was also negative for the presence of parasites through both direct and centrifugal-flotation methods. Culture and PCR tests conducted at UFMG confirmed the presence of *Clostridium perfringens* type A in the pre-death fecal sample of *Choloepus didactylus*, suggesting a possible etiological diagnosis of gastric dilatation and volvulus (GDV) in *C. didactylus*.

### 3.2. Post-Mortem Examination

**Figure 2:**
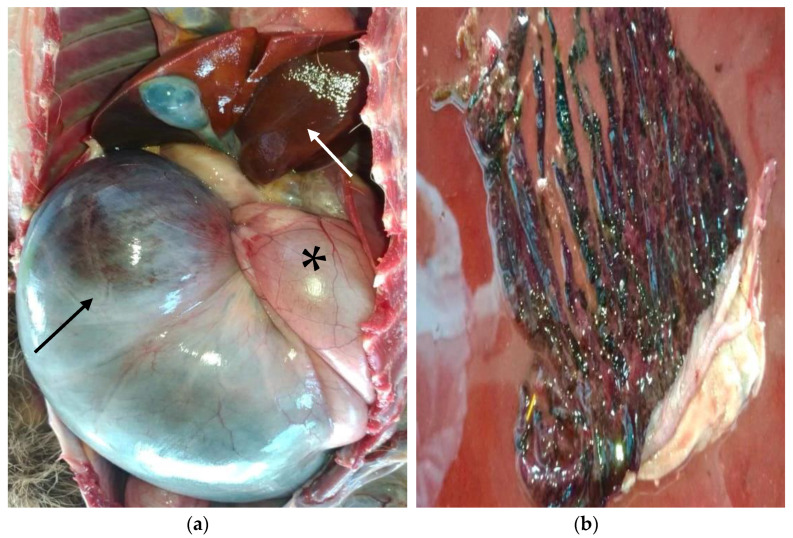
Macroscopic findings. (**a**) Distension of the squamous gastric compartment due to the presence of air (*), with a distended, purple–black glandular stomach (black arrow) and hepatic displacement (white arrow). (**b**) Severe hemorrhage and necrosis of the glandular stomach mucosa, accompanied by a large quantity of reddish liquid content.

A necropsy was performed on the same day the animal died, revealing a well-preserved carcass weighing 5.5 kg, with a good body condition score (3/5), a healthy amount of muscle mass, fat reserves, and moderate dehydration. The abdomen was markedly distended and tense. There was gas distension in the squamous gastric compartment (Figure 2a), with pink mucosa and a small amount of brown–green fluid with ingesta present. A volvulus of 180–270° was noted, occluding the gastroesophageal region, with a displacement of the visceral surface of the liver towards the right and cranial quadrant of the abdomen (Figure 2a) [18]. More than 70% of the mucosal surface of the glandular stomach appeared purplish black (Figure 2a), with severely hemorrhagic and necrotic mucosa containing a large amount of reddish fluid (Figure 2b).

Additionally, the duodenal portion of the intestine exhibited hyperemic mucosa with a yellow–greenish pasty content. The urinary bladder and colon were empty. The liver, brain, spleen, kidneys, lungs, and heart showed no significant findings. Samples of the stomach, lungs, liver, kidneys, spleen, adrenal glands, urinary bladder, heart, skeletal muscles, brain, and both small and large intestines were collected and immediately fixed in 10% neutral buffered formalin for histopathological examination. These samples were sent to the Animal Pathology Laboratory at UFPA, where they were routinely processed, embedded in paraffin, sectioned at 5 μm, and stained with hematoxylin and eosin (H&E) for microscopic analysis [19].

### 3.3. Histological Findings

**Figure 3:**
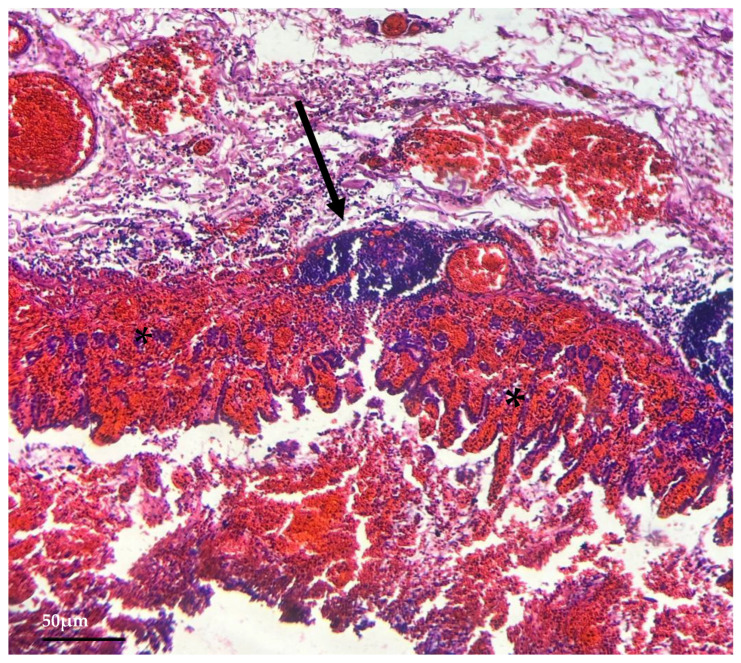
**Histopathological examination** (H&E obj. 10×). Marked congestion with mucosal hemorrhage and necrosis (*), along with the formation of tertiary lymphoid follicles (black arrow).

Histopathological examination revealed mild congestion in the non-glandular (squamous) portion of the stomach. In contrast, the glandular region showed marked congestion, hemorrhage, and mucosal necrosis (Figure 3), with denudation of the superficial layers and sloughed epithelial cells. Moderate mononuclear cell infiltrates, mainly lymphocytes, were observed in the superficial layers of the gastric submucosa, with the formation of tertiary lymphoid follicles (Figure 3). The spleen was diffusely congested with centrofollicular lymphoid depletion. No significant findings were observed in the liver, urinary bladder, lungs, heart, brain, duodenum, colon, kidneys, adrenal glands, or skeletal muscles.

## 4. Discussion

Most young sloths develop cases of constipation and bloat correlated with the stress of captivity and eventually die [3,5,8]. Mortality rates are higher in *Bradypus variegatus* (brown-throated sloth) individuals [3,8,11], but despite adapting better, Linnaeus’s two-toed sloth (LTT) is also susceptible [10]. Regarding GDV in sloths, there have been reports of three fatal cases occurring in LTT juveniles under 1 year of age in captivity at international institutions [10]. GDV is also frequently reported in humans, dogs, cats, pigs, and other non-domestic animals [20,21,22,23,24]. This case report describes an acute fatal GDV case in an adult LTT that had been in captivity for 5 years, marking the first case of this condition reported in this species in Brazil.

Studies have identified that pathological conditions in sloths were more frequent in younger animals with less than 7 months in captivity [3,8,18], similar to the three cases previously described in the literature. There is also a strong correlation between polytraumatized animals and the clinical presentation of gastrointestinal disorders [8], as well as the occurrence of stressful events prior to GDV development in dogs [19]. However, the patient in this report arrived at the veterinary hospital as an adult and, despite the severe trauma from electric shock, adapted very well to the daily management routine.

Over the 5 years in captivity, the sloth remained isolated most of the time with minimal human contact, being handled only for daily sunbathing and feeding, and was not kept on display. Occasionally, it exhibited episodes of abdominal distension, similar to bloat, which was immediately managed by administering simethicone, a common practice in the care of these animals in captivity, resulting in a positive response [8,25]. Thus, the long period in captivity and the sloth’s age in the present case, which differs from the three previously reported cases, further support the hypothesis that GDV may have multifactorial causes in sloths, as already reported for other species.

In contrast, GDV in dogs has been associated with older animals due to the loosening of the ligaments supporting the stomach [26]. However, a recent study indicates that the abdominal organ ligaments of the brown-throated sloth are strong enough to allow feeding in various positions without causing diaphragm compression or respiratory discomfort [27]. This behavior is attributed to the arboreal nature of sloths, which may also apply to LTTs. It raises the question of whether this could be a factor associated with the development of GDV in this species.

In addition to gas accumulation, gastric rotation results in obstruction of the esophageal and pyloric sphincters, with potentially severe physiological consequences. As the condition progresses, clinical signs compatible with shock may occur, such as tachycardia, weak pulse, prolonged capillary refill time, pale mucous membranes, hypothermia, tachypnea, and dyspnea [9,21,22,24]. In LTTs, the animals were diagnosed only post-mortem, similar to other non-domestic animals [10,20,22,23,24]. Two LTTs were found dead without premonitory clinical signs, while another presented with clinical signs consistent with bloat for 3 weeks before dying [10].

In sloths, GDV has also been correlated with prolonged periods of anorexia and low environmental temperatures [10]. In this report, the sloth showed signs of hyporexia the day before death without changes in urination and defecation frequency, and the next day, it was found with abdominal distension and clinical signs compatible with shock. This presentation is very similar to that in other animals [9,22], with the diagnosis made only post-mortem, as was the case in the three previously described LTT cases [10]. Although stabilization was attempted for later imaging and surgical intervention, as recommended for GDV cases [9,20,21,22,24], the patient died, demonstrating the rapidity and severity of the condition.

In this context, emergency treatment and rapid diagnosis of GDV cases are essential for stabilization and improved prognosis. Regarding ambient temperature, it was highly variable during the patient’s hospitalization. However, there was no sudden drop in temperature the day before that could justify the GDV episode. It is known that sloths are heterothermic and have limited thermoregulatory ability, making them sensitive to sudden temperature changes, which is an environmental and stress factor to consider in captive animals presenting with GDV [1,3].

The pathophysiology of GDV is complex; gastric distension and torsion produce both local and systemic effects, resulting in venous obstruction and significant oxygen supply reduction to areas such as the stomach, intestines, and spleen, potentially leading to ischemic necrosis and enterotoxemia [9]. Disseminated intravascular coagulation (DIC), systemic inflammatory response syndrome (SIRS), and multiple organ dysfunction syndrome (MODS) are some of the complications that can occur even after successful surgical correction [9]. Therefore, the post-mortem findings of hemorrhage in the gastric mucosa and submucosa, intestinal mucosal hyperemia, and splenic congestion are consistent with this pathogenesis and similar to the three previously reported cases, although no pulmonary changes were found in the present case, unlike in others [10].

The presence of mononuclear inflammatory cell infiltration in this report is similar to that described in a fatal case of gastric amebiasis in an LTT, indicating that it may be closely related to the intensity of the inflammatory response [7]. Conversely, GDV in sloths is also attributed to an inadequate diet [10]. The sloth’s diet in this report was similar to that described for other captive LTTs, with independent analyses ensuring it met the animal’s nutritional requirements. However, it was noted that the mashed food offered in the afternoon had fermented when removed, and the patient had a habit of geophagy during daily sunbathing sessions.

The ingestion of easily fermentable substrates, microbes capable of rapid fermentation, reflux of fermenting microbes from the duodenum to the stomach, dysbiosis, or a combination of these factors has been suggested as predisposing factors for acute gastric dilatation (AGD) in domestic and non-domestic animals, with volvulus suspected to follow AGD due to the rapid gas production by bacterial fermentation in the stomach [9,28]. Geophagy, reported in the literature as routine and poorly understood, may help digest toxins present in leaves or obtain dietary minerals [29,30].

There is a report of fatal gastritis associated with *Naegleria australiensis* infection in LTT [7], of *Sarcina* spp. as a presumptive cause of AGD in dogs, cats, horses, and as a cause of AGD and gastric emphysema in *Macaca mulatta* (rhesus monkeys) [31,32,33], and of *Clostridium perfringens* in non-domestic animals with AGD [28,34,35]. However, there is a lack of published research on infectious agents and clinical diseases in sloths [11]. In this study, *Clostridium perfringens* type A was the only pathogen found in fecal analyses collected two days before death.

The authors suggest that the presence of *C. perfringens*, an anaerobic, gas-producing bacillus commonly found in the environment, in the stomach and feces may contribute to the development of AGD and GDV [28,34,35]. In domestic animals, diet and environmental changes can predispose to clostridial diseases. The LTT’s gastrointestinal microbiome is highly specialized, dominated by bacteria from the phyla Proteobacteria and Firmicutes, without Clostridiaceae. Therefore, *C. perfringens* would not be part of the normal fecal community of LTT [4].

However, various factors can influence the microbiota composition, such as diet and the environment in which the animals live [4,5]. In this case, the diet offered, which had a large amount of soluble and easily fermented carbohydrates, such as fruits and broccoli, and the geophagy habit may have been sources of multiplication and contamination by the bacteria. Even though the diet and the stomach were not tested, it is believed that *C. perfringens* may have played a significant role as a cause or contributor to the intensity, severity, and rapid progression of the condition. It should be considered in future studies on the etiology of AGD and GDV in sloths, with gastric bacterial culture recommended in future studies. Therefore, further studies are necessary to correlate the presence of this bacterial agent with the occurrence of GDV in sloths, as well as to determine the epidemiology and prevalence of GDV in captive sloths and establish preventive and treatment protocols.

## 5. Conclusions

This report describes the first documented case of acute and fatal gastric dilatation and volvulus (GDV) in an adult captive Linnaeus’s two-toed sloth (*Choloepus didactylus*) in Brazil. Hyporexia was the clinical sign observed the day before the sloth’s death, with subsequent signs indicating shock. GDV was diagnosed post-mortem, and fecal samples collected two days before death were positive for *Clostridium perfringens type* A. GDV should be considered as a potential cause of sudden death in adult captive sloths. Although GDV has a multifactorial etiology, *C. perfringens* must be included in the differential diagnosis. Future research should focus on understanding the multifactorial causes of GDV in sloths, particularly the role of gut microbiota and pathogens like *C. perfringens*, to improve preventive measures in captive settings.

## Figures and Tables

**Table 1:** Fixed diet provided to the sloth (*C. didactylus*) in the morning and afternoon at WSVT IVM/UFPA.

	Food Item Quantity	Food Item Quantity
**Morning Period (food provided in strips)**	Apple	20 g
	Melon	20 g
	Carrot	20 g
	Boiled chicken egg (shell removed)	20 g
**Afternoon (soft food)**	Banana	40 g
	Papaya	35 g
	Apple	20 g
	Carrot	35 g
	Broccoli	20 g
	Chicken	30 g
	Boiled egg (shell removed)	½
	Water	150 mL

## Data Availability

Data are contained within the article.
